# Differential pulse oximetry readings between ethnic groups and delayed transfer to intensive care units

**DOI:** 10.1093/qjmed/hcac218

**Published:** 2022-09-06

**Authors:** C J Crooks, J West, J R Morling, M Simmonds, I Juurlink, S Briggs, S Cruickshank, S Hammond-Pears, D Shaw, T R Card, A W Fogarty

**Affiliations:** Nottingham Digestive Diseases Centre, School of Medicine, University of Nottingham, Nottingham NG7 2UH, UK; NIHR Nottingham Biomedical Research Centre (BRC), Nottingham University Hospitals NHS Trust and the University of Nottingham, Nottingham NG7 2UH, UK; Nottingham University Hospitals NHS Trust, Nottingham NG7 2UH, UK; NIHR Nottingham Biomedical Research Centre (BRC), Nottingham University Hospitals NHS Trust and the University of Nottingham, Nottingham NG7 2UH, UK; Nottingham University Hospitals NHS Trust, Nottingham NG7 2UH, UK; Lifespan and Population Health, School of Medicine, University of Nottingham, Nottingham NG5 1PB, UK; East Midlands Academic Health Science Network, University of Nottingham, Nottingham NG7 2TU, UK; NIHR Nottingham Biomedical Research Centre (BRC), Nottingham University Hospitals NHS Trust and the University of Nottingham, Nottingham NG7 2UH, UK; Nottingham University Hospitals NHS Trust, Nottingham NG7 2UH, UK; Lifespan and Population Health, School of Medicine, University of Nottingham, Nottingham NG5 1PB, UK; Nottingham University Hospitals NHS Trust, Nottingham NG7 2UH, UK; Nottingham University Hospitals NHS Trust, Nottingham NG7 2UH, UK; Nottingham University Hospitals NHS Trust, Nottingham NG7 2UH, UK; Nottingham University Hospitals NHS Trust, Nottingham NG7 2UH, UK; Nottingham University Hospitals NHS Trust, Nottingham NG7 2UH, UK; East Midlands Academic Health Science Network, University of Nottingham, Nottingham NG7 2TU, UK; Nottingham University Hospitals NHS Trust, Nottingham NG7 2UH, UK; Division of Respiratory Medicine, School of Medicine, University of Nottingham, Nottingham NG5 1PB, UK; NIHR Nottingham Biomedical Research Centre (BRC), Nottingham University Hospitals NHS Trust and the University of Nottingham, Nottingham NG7 2UH, UK; Nottingham University Hospitals NHS Trust, Nottingham NG7 2UH, UK; Lifespan and Population Health, School of Medicine, University of Nottingham, Nottingham NG5 1PB, UK; NIHR Nottingham Biomedical Research Centre (BRC), Nottingham University Hospitals NHS Trust and the University of Nottingham, Nottingham NG7 2UH, UK; Nottingham University Hospitals NHS Trust, Nottingham NG7 2UH, UK; Lifespan and Population Health, School of Medicine, University of Nottingham, Nottingham NG5 1PB, UK

## Abstract

**Background:**

Pulse oximeters are widely used to monitor blood oxygen saturations, although concerns exist that they are less accurate in individuals with pigmented skin.

**Aims:**

This study aimed to determine if patients with pigmented skin were more severely unwell at the period of transfer to intensive care units (ICUs) than individuals with White skin.

**Methods:**

Using data from a large teaching hospital, measures of clinical severity at the time of transfer of patients with COVID-19 infection to ICUs were assessed, and how this varied by ethnic group.

**Results:**

Data were available on 748 adults. Median pulse oximetry demonstrated similar oxygen saturations at the time of transfer to ICUs (Kruskal–Wallis test, *P* = 0.51), although median oxygen saturation measurements from arterial blood gases at this time demonstrated lower oxygen saturations in patients classified as Indian/Pakistani ethnicity (91.6%) and Black/Mixed ethnicity (93.0%), compared to those classified as a White ethnicity (94.4%, Kruskal–Wallis test, *P* = 0.005). There were significant differences in mean respiratory rates in these patients (*P* < 0.0001), ranging from 26 breaths/min in individuals with White ethnicity to 30 breaths/min for those classified as Indian/Pakistani ethnicity and 31 for those who were classified as Black/Mixed ethnicity.

**Conclusions:**

These data are consistent with the hypothesis that differential measurement error for pulse oximeter readings negatively impact on the escalation of clinical care in individuals from other than White ethnic groups. This has implications for healthcare in Africa and South-East Asia and may contribute to differences in health outcomes across ethnic groups globally.

## Introduction

Pulse oximetry is the main method of monitoring oxygen saturation in clinical settings including pre-hospital evaluation, to outpatient clinics, inpatients on hospital wards and intensive care units (ICUs). The method uses two light beams of different frequencies, and the fact that the differential light absorptions of these light beams vary according to blood oxygen saturation.^1^ It is convenient and allows continual measurements to be made, in a non-invasive manner.

However, in 1990, it was reported that pulse oximeters may have a differential measurement error in individuals with pigmented skin, presumably as a consequence of the pigmented skin resulting in differential absorption of the light as it passes through translucent tissue.^2^ This was followed by a similar report in 2020,^3^ which led to a notification of concern by the Food and Drink Administration in the USA stating that pulse oximetry ‘may be less accurate in people with dark skin pigmentation’.^4^ The UK Medicine and Healthcare Regulatory Products Agency states that they are ‘not aware of any incidents where skin color has had an adverse effect on the use of pulse oximeters when providing effective healthcare’,^5^ and it may be challenging to identify such cases at the individual clinical level, as many factors contribute to clinical decision-making and outcomes. However, if the measurement error is such that patients with pigmented skin have higher oxygen saturation measurements from the pulse oximetry readings than those generated by the gold standard direct measurement of arterial blood gas, this may thus provide a misleadingly positive clinical scenario which may then delay escalation of care in these patients.

The COVID-19 pandemic has resulted in repeated surges of admissions of patients with respiratory failure who require intensive care support. Patients from ethnic groups with darker skin color have markedly higher mortality from COVID-19 infection, which is not explained by known comorbidities, social factors or lifestyle indices suggesting that unknown issues are contributing to these ethnic disparities.^6^ Pulse oximetry is one of the main tools used to monitor these patients, and contributes to the decision-making process and timing of when a patient’s care is escalated to ICUs for more invasive respiratory and other organ support.

Having previously demonstrated that pulse oximetry readings in individuals with pigmented skin are providing erroneously higher measures of oxygen saturation in patients outside of intensive care with COVID-19 infection,^7^ this study now focuses on the physiological differences at the time of admission to intensive care associated with ethnicity, as any differences between ethnic groups may be a consequence of differences in awareness of disease severity secondary to the pulse oximetry measurement error. We hypothesize that in patients from ethnic groups with greater measurement error of pulse oximetry there will be greater delay, and so patients will be sicker with higher respiratory rates at the time of ICU admission, despite similar pulse oximetry readings just prior to transfer to ICUs. We tested this hypothesis using data from a cohort of all patients with COVID-19 infection admitted to ICUs in a busy teaching hospital.

## Methods

We conducted a retrospective cohort study using routinely collected electronic data for patients admitted to a level 3 intensive treatment unit bed at Nottingham University Hospitals NHS trust between 1 February 2020 and 24 November 2021 with COVID-19 infection. The last recorded observations and blood tests were extracted prior to the time of ITU admission. A detailed description of the full cohort is available.^8^ Oxygen saturations from ward oximetry measurements are recorded routinely electronically using NerveCentre (http://nervecentresoftware.com/), and arterial blood gas measurements are automatically uploaded to the hospital enterprise data warehouse. Dates for COVID-19-related vaccinations were available within the anonymized secondary care patient records via a link from their primary care records.

Median and interquartile range (IQR) for oxygen saturations by both pulse oximetry and arterial blood gas, fraction of inspired oxygen, respiratory rate, systolic blood pressure, heart rate and temperature were stratified by recorded ethnicity of White, Mixed, Asian, Black and Other Ethnic group (please see the Appendix for how Ethnic Groups are categorized by the UK Government). Patients with unknown ethnicity or without a recorded ethnicity were initially treated as a separate category to present the data in its entirety but excluded from multivariate analysis.

Finally, a linear model predicting each measurement by ethnicity was fitted, adjusted for age (<50, 51–70, 71–80 and 81–90) and sex.

All analyses were performed using version 4.1.2 of the R programming language (R project for Statistical Computing; R Foundation).

Approval for this work was granted via an NUH Clinical Effectiveness Team audit (reference: reference: 21-294C) and IRAS (REC: 20/WM/0142, project ID: 282490, amendment No. SA02 20/07/21).

## Results

Data were available on 748 adults prior to their transfer to ICUs and 420 (56%) were classified as being of White ethnicity (Table 1). The mean age was 56 years, and the mean age at time of transfer to ICUs varied across ethnic groups (*P* < 0.001), being lowest in those of ‘Other’ ethnicity with a mean value of 49 years, and highest in those of White ethnicity with a mean value of 58 years. Ten percent or less of patients requiring ICU admission had received two vaccination doses at the time of their COVID-19 infection.

**Table 1.** Description of physiological status of patients with COVID-19 infection who were transferred to intensive care units stratified by ethnic group

Ethnicity	White	Unrecorded	Black/mixed	Indian/Pakistani	Other	*P*-value
**No of patients, (%)**	**420**	**205**	**48**	**53**	**22**	<0.00001^a^
**Male sex (%)**	**61**	**69**	**52**	**64**	**50**	0.09^a^
**Mean age, years,** **(95% CI)**	**58** **(27–89)**	**56** **(27–85)**	**52** **(27–76)**	**51** **(23–79)**	**49** **(25–72)**	**0.0002** ^b^
**First vaccination dose only >14 days before COVID-19 (%)**	**4**	**2**	**2**	**6**	**0**	
**Second vaccination dose >14 days before COVID-19 (%)**	**10**	**7**	**2**	**6**	**0**	**0.22** ^a^
**Weekend ICU admission (%)**	**25**	**31**	**31**	**23**	**45**	**0.14^a^**
**Median pulse oximetry, O_2_% (IQR)**	**94 (92–96)**	**94 (91–96)**	**94 (91–95)**	**94 (92–96)**	**94 (89–95)**	**0.51** ^c^
Missing (n)	65 (15%)	34 (17%)	4 (8%)	6 (11%)	4 (18%)	
**Median blood gas—oxygen, O_2_% (IQR)**	**94.4** **(90.4–97.5)**	**94.2** **(90.2–97.3)**	**93.0** **(89.8–95.5)**	**91.6** **(89.3–94.4)**	**92.0** **(89.3–94.8)**	**0.005** ^c^
Missing (*n*)	96 (23%)	61 (30%)	13 (27%)	12 (23%)	7 (32%)	
**Median inspired O_2_, %, (IQR)**	**60 (40–95)**	**60 (35–75)**	**65 (54–85)**	**60 (40–90)**	**70 (52–95)**	**0.09** ^c^
Missing (*n*)	199 (47%)	122 (60%)	28 (58%)	28 (53%)	8 (36%)	
**Mean respiratory rate, per min, (95% CI)**	**26 (8–43)**	**29 (10–47)**	**31 (13–48)**	**30 (16–43)**	**31 (17–45)**	**<0.00001** ^b^
Missing (*n*)	61 (15%)	29 (14%)	4 (8%)	6 (11%)	3 (14%)	
**Mean systolic BP, mmHg, (95% CI)**	**128** **(79–177)**	**128** **(80–175)**	**133** **(79–187)**	**124** **(89–159)**	**132** **(93–170)**	**0.42** ^b^
Missing (*n*)	65 (15%)	33 (16%)	4 (8%)	6 (11%)	4 (18%)	
**Mean heart rate, per min, (95% CI)**	**93** **(51–135)**	**95** **(53–137)**	**95** **(54–136)**	**95** **(62–128)**	**96** **(63–128)**	**0.61** ^b^
Missing (*n*)	64 (15%)	32 (16%)	4 (8%)	6 (11%)	4 (18%)	
**Mean temp,** **°C, (95% CI)**	**37 (35–39)**	**37 (36–39)**	**37 (36–38)**	**37 (35–38)**	**37 (36–38)**	**0.5^b^**
Missing (*n*)	64 (15%)	29 (14%)	4 (8%)	6 (11%)	3 (14%)	

a Chi-squared test.

b ANOVA statistical test.

c Kruskal–Wallis test.

CI, 95% confidence interval; BP, blood pressure; Temp, temperature.

There were similar oxygen saturations at the time of transfer to ICUs as measured by pulse oximetry (see Table 1) (Kruskal–Wallis test, *P* = 0.51), but not as measured by arterial blood gases (Kruskal–Wallis test, *P* = 0.005), with the highest median measures in those classified as being of a White ethnicity (94.4%, IQR: 90.4–97.5) and the lowest in individuals from an Indian/Pakistani ethnicity (91.6%; IQR: 89.3–94.4). However, there were significant differences in mean respiratory rates in these patients (*P* < 0.0001), ranging from 26 breaths/min in individuals with White ethnicity to 30 for those who were classified as Indian/Pakistani ethnicity and 31 breaths/min for those classified as Black/Mixed/Other ethnicity. (Table 2 These differences persisted after adjustment for age, sex, weekend ICU admission, and vaccination status, with individuals with an ethnicity classified as ‘Other than White’ having a respiratory rate of 3.5 breaths/minute (95% confidence intervals CI: 1.6 to 5.4) higher than those classified as White (Table 2). A similar adjusted analysis using different categories of ethnicity demonstrated higher respiratory rates in those of Indian/Pakistani ethnicity (+3.1 breaths per minute; 95% confidence intervals: +0.4 to +5.7) and Black/Mixed ethnicity (+3.9 breaths/minute; 95% CI: 1.2 to 6.7) compared to those classified as White ethnicity (Table 3).

**Table 2.** Multivariate linear regression analysis of respiratory rate of patients with COVID-19 infection prior to transfer to the intensive care unit stratified by White and Other than White ethnic groups (*N* = 469)

	Adjusted difference in respiratory rate	*P*-value
Ethnic group		
White	0.0 (reference)	<0.001
Other than White	+3.5 (+1.6 to +5.4)	
Age (years)		
<50	0.0 (reference)	
51–70	−1.6 (−3.4 to +0.23)	0.09
71–80	−3.9 (−6.3 to −1.6)	0.001
81–90	−9.9 (−14.4 to −5.4)	<0.001
Sex		
Male	0.0 (reference)	
Female	+0.16 (−1.43 to +1.76)	0.84
Weekend ICU admission		
No	0.0 (reference)	
Yes	+0.99 (−0.82 to 2.8)	0.28
Vaccination status >14 days before COVID-19 infection		
No vaccination	0.0 (reference)	
1st dose only	−2.9 (−7.2 to +1.4)	0.18
2nd dose	+2.7 (−0.11 to +5.5)	0.060

**Table 3.** Multivariate analysis of respiratory rate of patients with COVID-19 infection prior to transfer to the intensive care unit stratified by ethnic group (*N* = 469)

	Adjusted difference in respiratory rate	*P*-value
Ethnic group		
White	0.0 (reference)	
Indian/Pakistani	+3.1 (+0.4 to +5.7)	0.025
Black/Mixed	+3.9 (+1.2 to +6.7)	0.005
Other	+3.6 (−0.5 to +7.6)	0.08
Age (years)		
<50	0.0 (reference)	
51–70	−1.6 (−3.4 to +0.2)	0.08
71–80	−3.9 (−6.3 to −1.6)	0.001
81–90	−9.9 (−14.4 to −5.4)	<0.001
Sex		
Male	0.0(reference)	
Female	+0.1 (−1.5 to +1.7)	0.87
Weekend ICU admission		
False	0.0 (reference)	
True	+0.97 (−0.85 to 2.8)	0.30
Vaccination status >14 days before COVID-19 infection		
No vaccination	0.0 (reference)	
1st dose only	−2.9 (−7.2 to +1.5)	0.19
2nd dose	+2.7 (−0.11 to +5.5)	0.060

There was no difference in fractional inspired oxygen, systolic blood pressure, heart rate or temperature between these ethnic groups.

## Discussion

These data represent the first analysis to explore the hypothesis that differential measurement error across ethnic groups may be associated with more severe respiratory failure at the time of transfer to ICUs. The results demonstrate that individuals from ethnic groups with non-white skin color have higher respiratory rates at the point of transfer to intensive care compared to those with white skin color, and thus are likely to have more advanced respiratory failure at this time period. Despite having a higher respiratory rate, individuals with non-white skin color still had lower oxygen saturations as measured by arterial blood gases, although identical oxygen saturations as measured by pulse oximetry.

The strengths of these data are that they are from a complete dataset of all patients with COVID-19 infection who were admitted to a large teaching hospital during the COVID-19 pandemic, and that the electronic data collection system allows the observations of vital signs to be archived and used in this analysis. The data are from groups of patients who all had the same infection with the COVID-19 virus, thus providing the same disease process to be compared across ethnic groups, and reducing the risk of confounding by differential disease processes. The clinical data used to inform the decision as to the timing of transfer to ICUs is presented in Table 1, and it can be seen that the main difference between ethnic groups is a higher respiratory rate in those from ethnic groups with non-white skin pigmentation, with no differences in oxygen saturation as measured by pulse oximetry, blood pressure, heart rate or temperature. This strongly suggests that individuals from the Other than White ethnic groups have more advanced respiratory failure at the time of transfer to intensive care which is supported by the finding of a lower mean blood gas PaO_2_.

The use of multivariate regression allows us to adjust for both age and sex, and be confident that this is not a consequence of residual confounding by these factors. The presence of higher respiratory rates in individuals with Black/Mixed ethnic groups compared to individuals from Indian/Pakistani ethnic groups relative to the White reference group hints at a dose–response in individuals with darker skin, although optimally objective measurements of skin pigmentation would be required to clarify this. A dose–response is one of the epidemiological criteria suggesting that a causal relationship may be present.^9^ Observing statistical differences in small numbers of patients suggests large differences between ethnic groups, as the analysis uses routinely collected categorical data on ethnic group for health service monitoring which takes no consideration of degree of skin pigmentation and is likely to contain wide variation in skin pigmentation within individual categories’ leading to random measurement error and loss of statistical power.

One potential limitation of these data is that the respiratory rate data is collected by subjective measurement, and hence there is a theoretical risk of differential measurement error by ethnic group. However, this is very unlikely, as most of the patients transferred to ICUs were cared for by experienced emergency department or respiratory nurses. While there may always be some random measurement error in subjective clinical measurements, it is difficult to envisage a clinical scenario where this can occur to such a large extent in a systemic manner to account for these observations across different ethnic groups.

The observation that individuals from Other than White ethnic groups have more severe respiratory failure at the time of referral to ICUs than White ethnic groups is consistent with current knowledge of how pulse oximeters give an erroneously high measurement of oxygen saturation in the former groups compared to the latter. These observations were first reported in 1990, when it was noted that achieving satisfactory blood oxygenation as measured by arterial blood gases required a target pulse oximetry saturation of 92% in White patients, and 95% in Black patients.^2^ Subsequently in 2005, an observational study in healthy volunteers reported that pulse oximetry gave readings that ranged from 2.3% to 4.3% higher than the real value in Black individuals compared to White individuals.^10^

These relatively small scale studies were followed by a larger analysis of 46 259 patients which explored the prevalence of ‘occult hypoxaemia’ as defined by a true oxygen saturation of less than 88% as measured by arterial blood gas in the context of a pulse oximetry reading of 92% or more. 17.0% of patients with black skin have ‘occult hypoxaemia’ compared to 6.2% of White patients.^3^ A recent observational study reported similar results, and also suggested that this was associated with delays to initiating treatment.^11^ We have recently completed a similar analysis in a population of COVID-19 patients, and observed that for individuals of an Other than White ethnicity with an arterial blood gas oxygen saturation in the clinically critical 85–89% range, the mean paired measurement from pulse oximetry was 5.4% higher, while the comparable measurement for those of a White ethnicity was +2.4%.^7^ Taken collectively, the later referral to intensive care in the ‘other than White’ ethnic groups is likely to be caused at least in part by the biased clinical information provided by pulse oximetry measurements. A further analysis suggests that this bias continues once the patient has transferred to ICUs, as individuals with non-white skin had less supplemental oxygen than those with white skin.^12^

If pulse oximeters provide erroneously higher oxygen saturations in individuals with pigmented skin then the implications are manifold. This may contribute to the worse health outcomes for individuals from ethnic groups with non-white skin for a variety of scenarios where timely clinical assessment and interventions are important. These include asthma,^13^ surgical procedures in children^14^ and adults^15^^,^^16^ and admission to intensive care,^17^ all of which will use pulse oximetry at some stage in the process for patient assessment and monitoring. COVID-19 infection itself is associated with a higher risk of mortality in those from ethnic groups with non-white skin,^18^ and differentials in initial clinical evaluation, decisions to admit to hospital and escalate medical treatment that were due to reassuringly higher pulse oximetry readings in these groups may have contributed to these outcomes.

In the short term, clinicians need to be aware of the risk of differential measurement error in individuals with pigmented skin and use respiratory rate as a primary tool to assess respiratory failure, supplemented by a lower threshold for measuring blood oxygen saturation directly by arterial blood gas sampling. In the longer term, better non-invasive methodologies to measure oxygen saturation are required in all ethnic groups, not just those with white skin. This is a global public health issue, as the United National estimates that 1.3 billion people live in Africa, and 4.7 billion people live in Asia,^19^ many of whom will have pigmented skin that can be described as ‘other than white’. These populations may be at risk of systemic measurement error from pulse oximeters impairing clinical decision-making.

## Funding

This work was funded by Nottingham University Hospitals NHS Trust and the University of Nottingham. Nottingham University Hospitals NHS Trust also sponsored the study. The funders had no role in the study execution and reporting.

*Conflict of interest*: None declared.

## Appendix: Categorisation of Ethnic Groups using UK Government methodology from https://www.ethnicity-facts-figures.service.gov.uk/style-guide/ethnic-groups

**White**

- English, Welsh, Scottish, Northern Irish or British
- Irish
- Gypsy or Irish Traveller
- Any other White background

**Mixed or Multiple ethnic groups**

- White and Black Caribbean
- White and Black African
- White and Asian
- Any other Mixed or Multiple ethnic background

**Asian or Asian British**

- Indian
- Pakistani
- Bangladeshi
- Chinese
- Any other Asian background

**Black, African, Caribbean or Black British**

- African
- Caribbean
- Any other Black, African or Caribbean background

**Other ethnic group**

- Arab
- Any other ethnic group
